# Supplementary figures and images for: DGQR estimation for interval censored quantile regression with varying-coefficient models
Source: PLoS One. 2020 Nov 10;15(11):e0240046. doi: 10.1371/journal.pone.0240046 (PMC7654815; doi:10.1371/journal.pone.0240046)

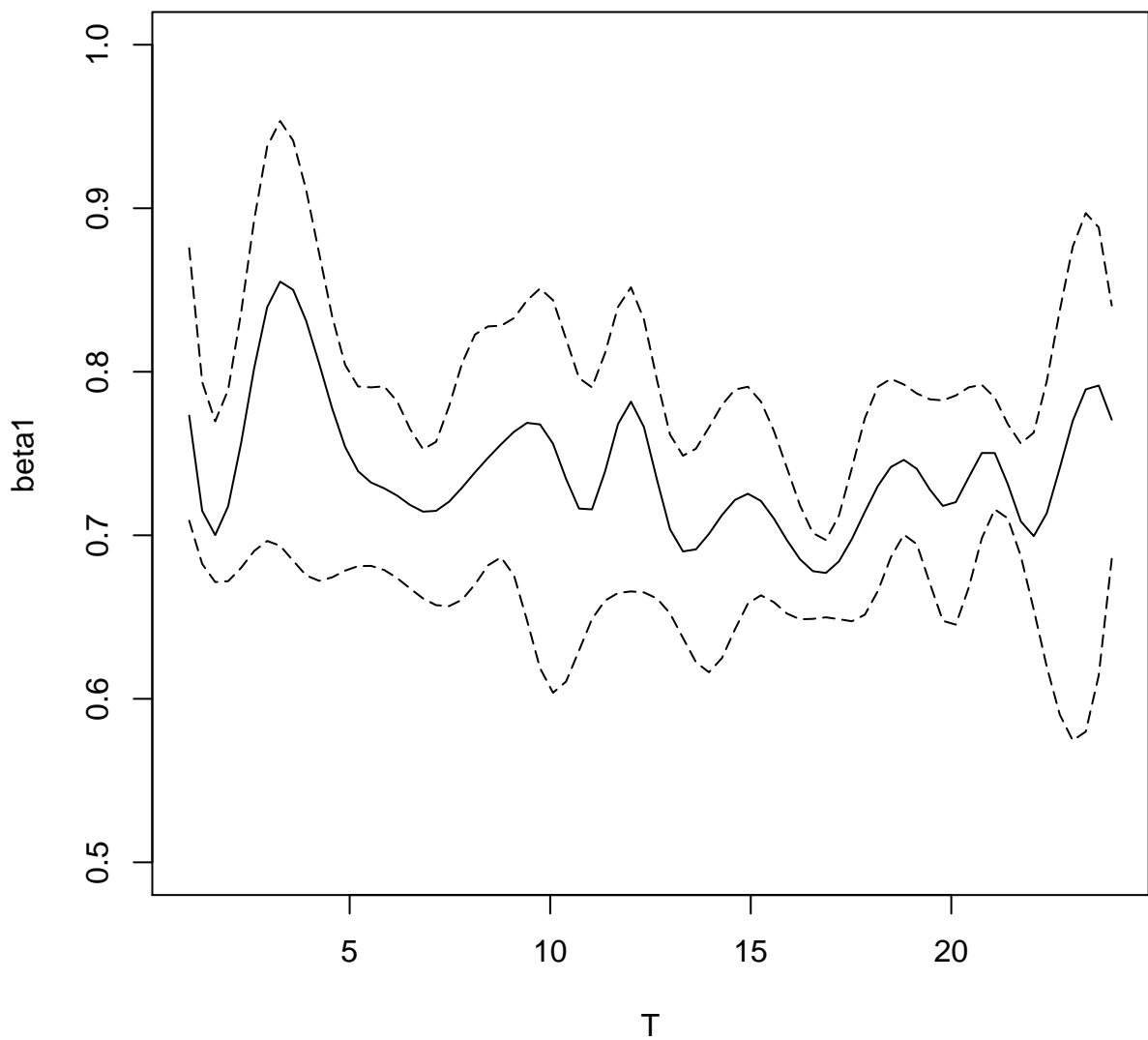

Supplement: S1 File — (ZIP) [file pone.0240046.s001.zip › DGQR estimation/beta1_censored.pdf]

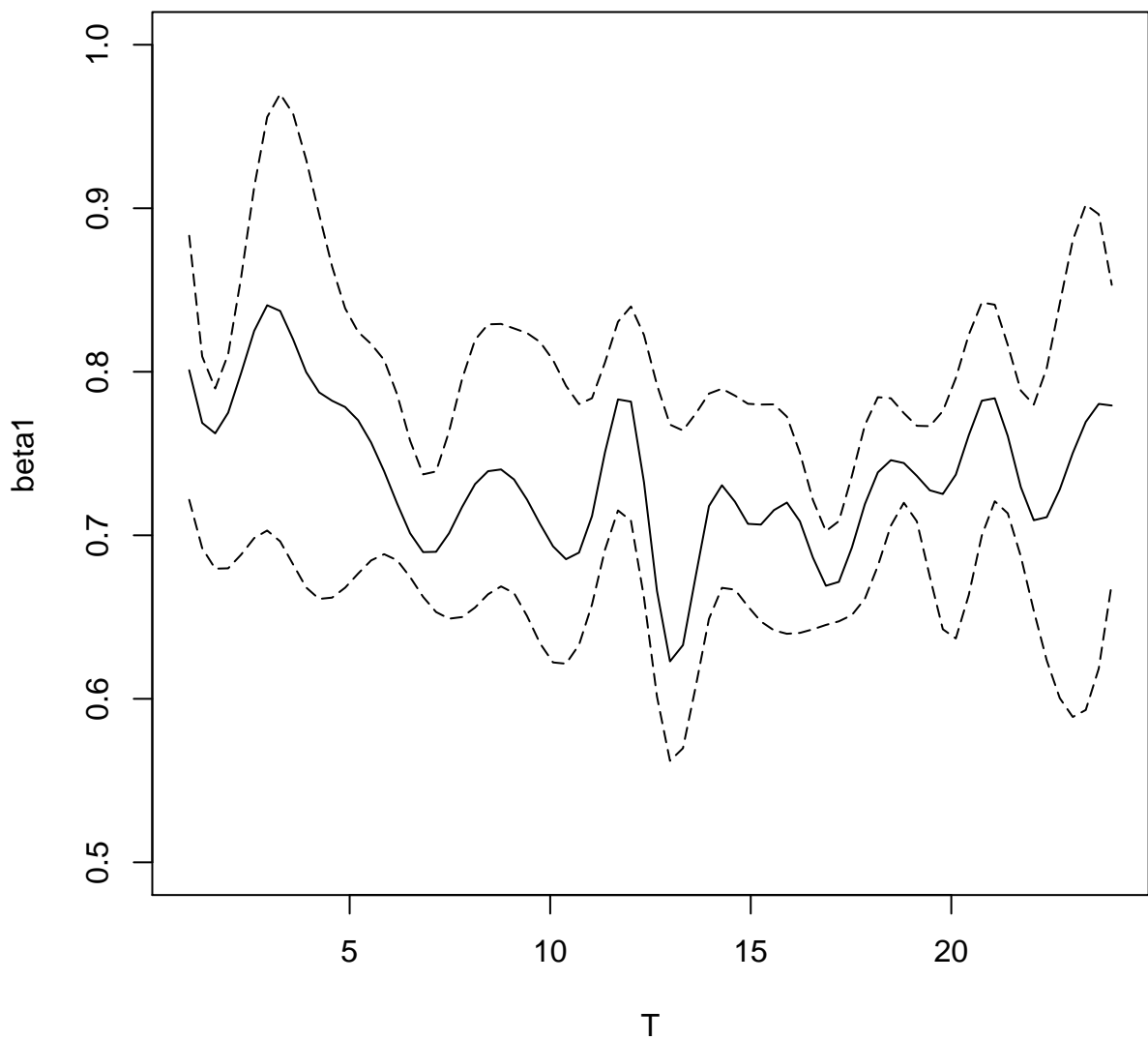

Supplement: S1 File — (ZIP) [file pone.0240046.s001.zip › DGQR estimation/beta1_complete.pdf]

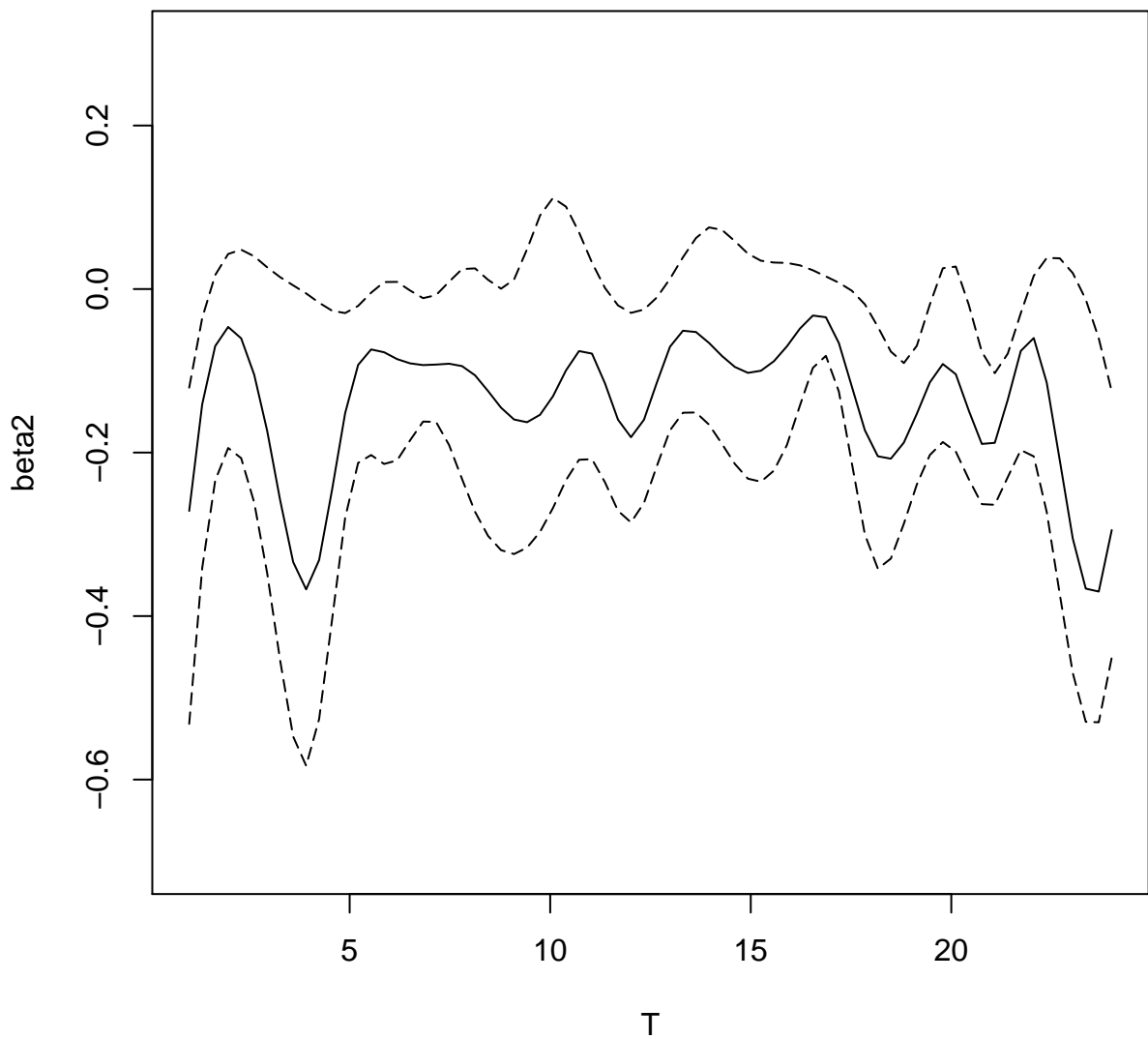

Supplement: S1 File — (ZIP) [file pone.0240046.s001.zip › DGQR estimation/beta2_censored.pdf]

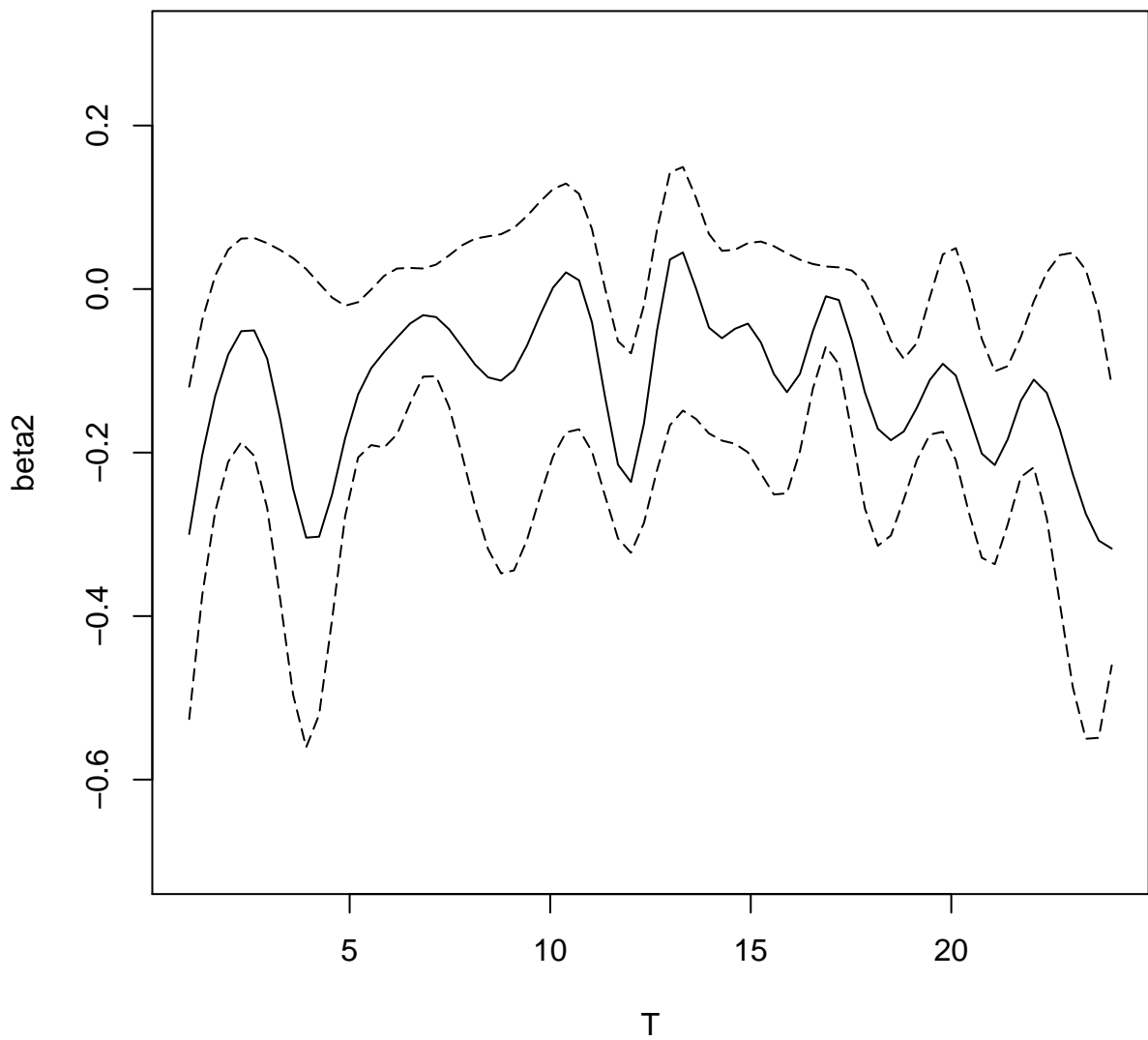

Supplement: S1 File — (ZIP) [file pone.0240046.s001.zip › DGQR estimation/beta2_complete.pdf]

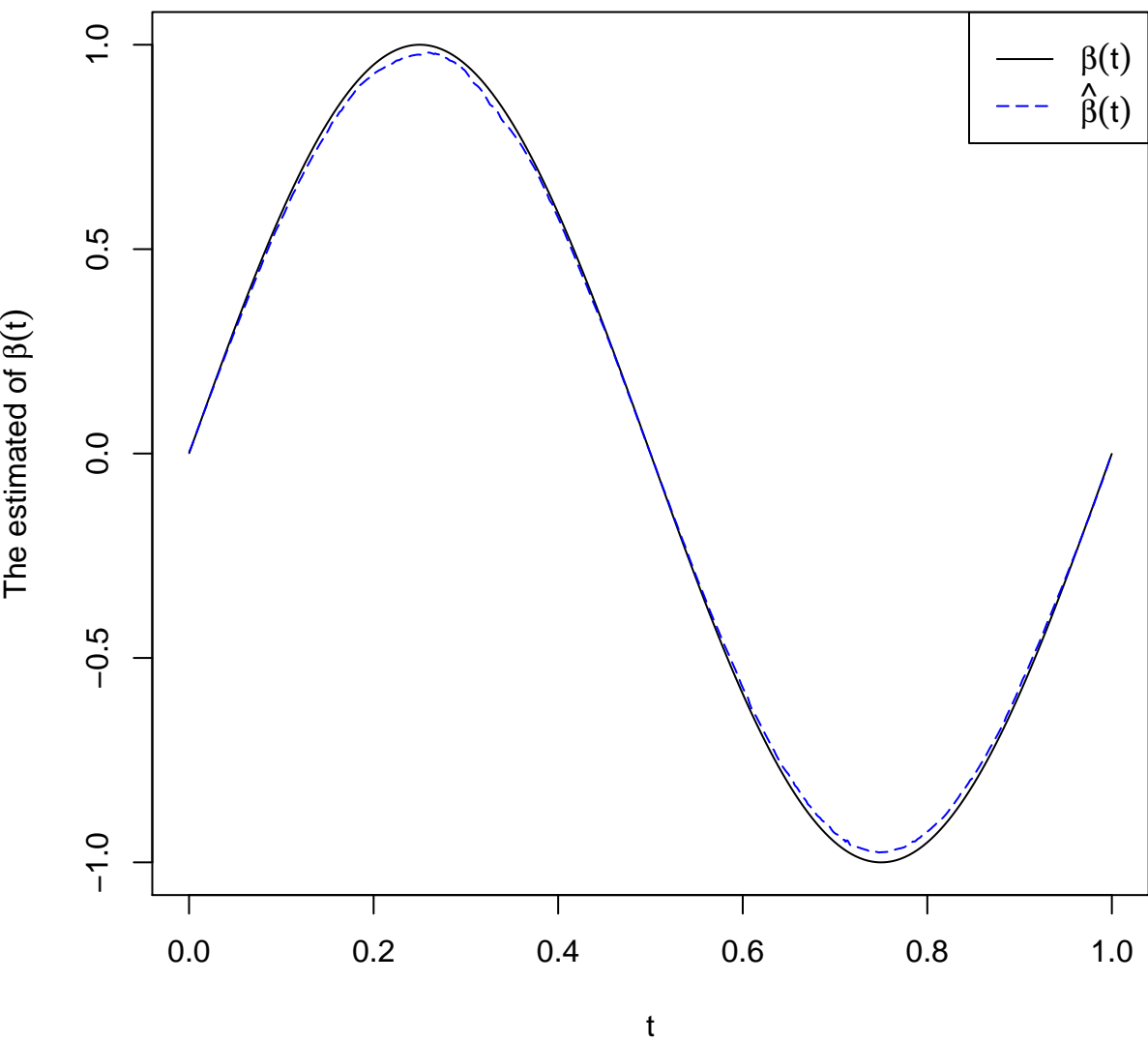

Supplement: S1 File — (ZIP) [file pone.0240046.s001.zip › DGQR estimation/example1.pdf]

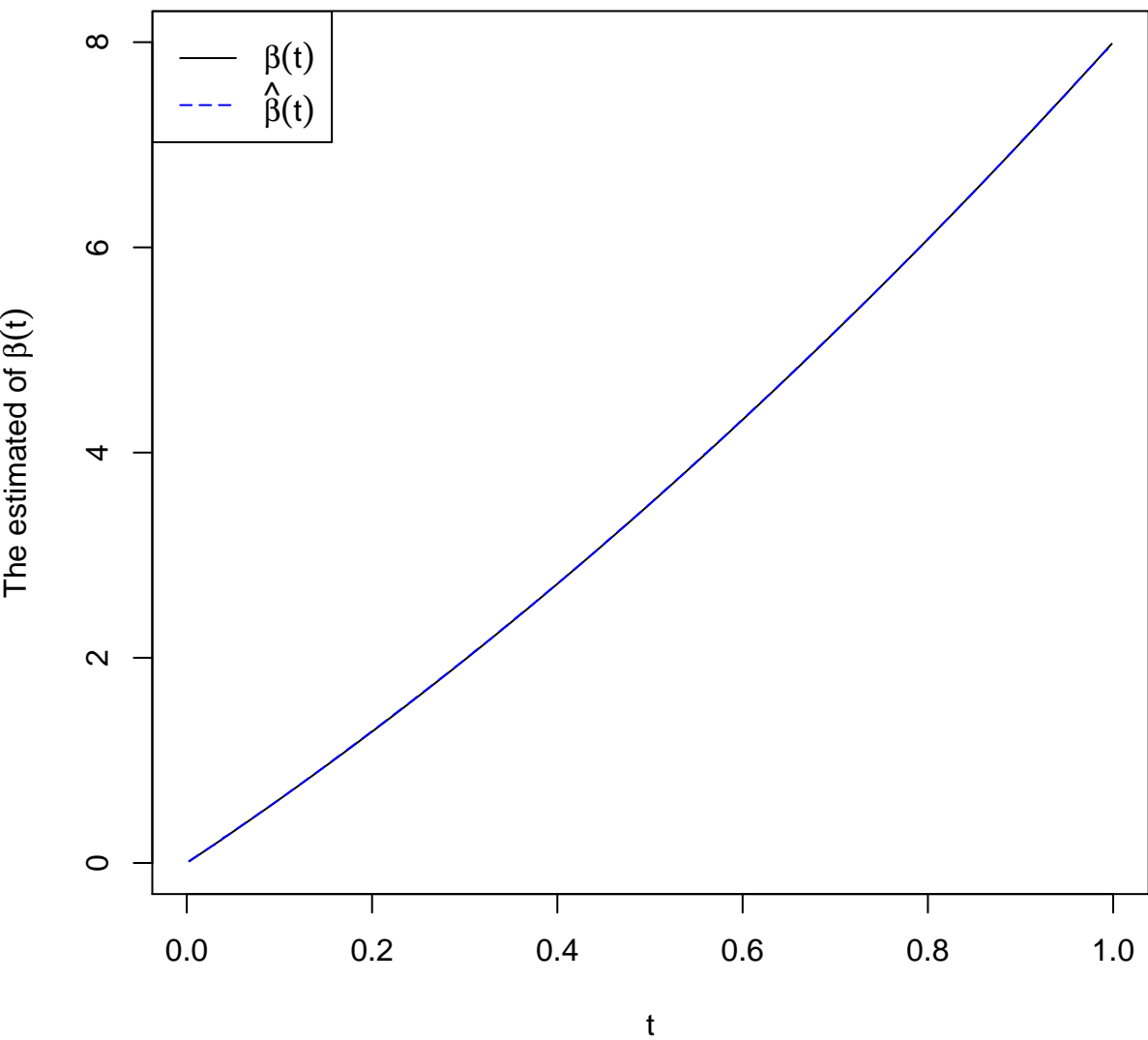

Supplement: S1 File — (ZIP) [file pone.0240046.s001.zip › DGQR estimation/example2.pdf]

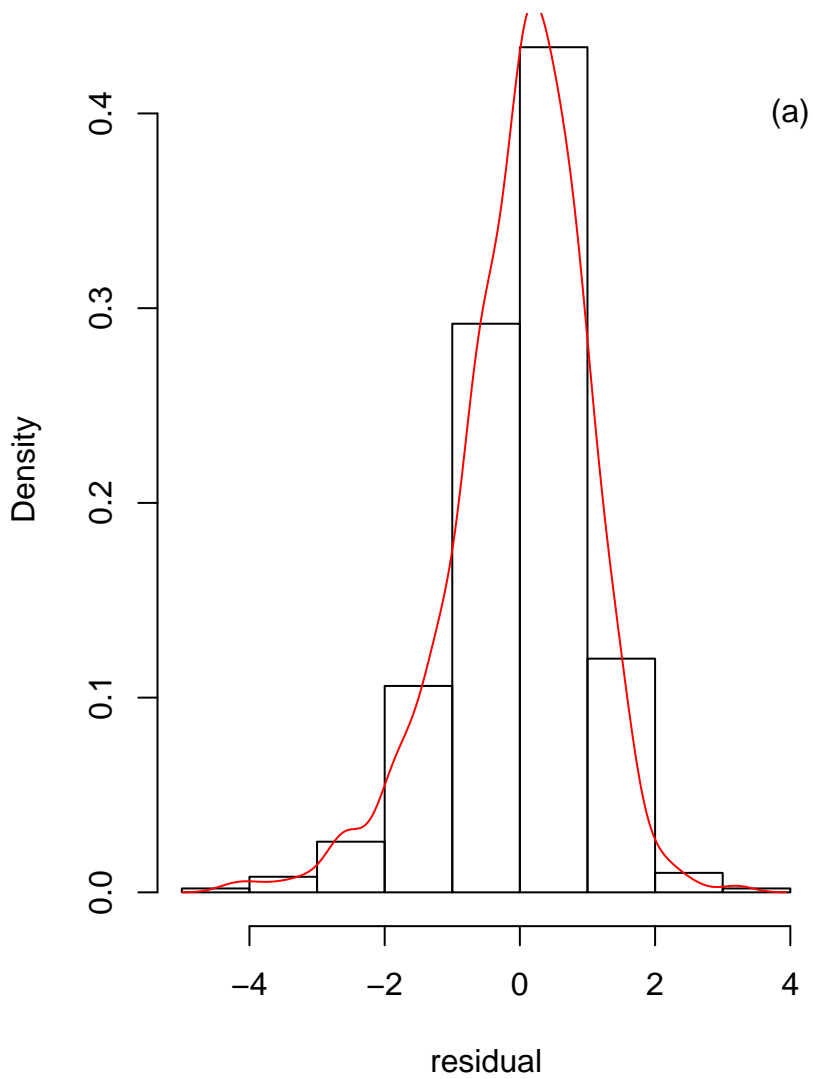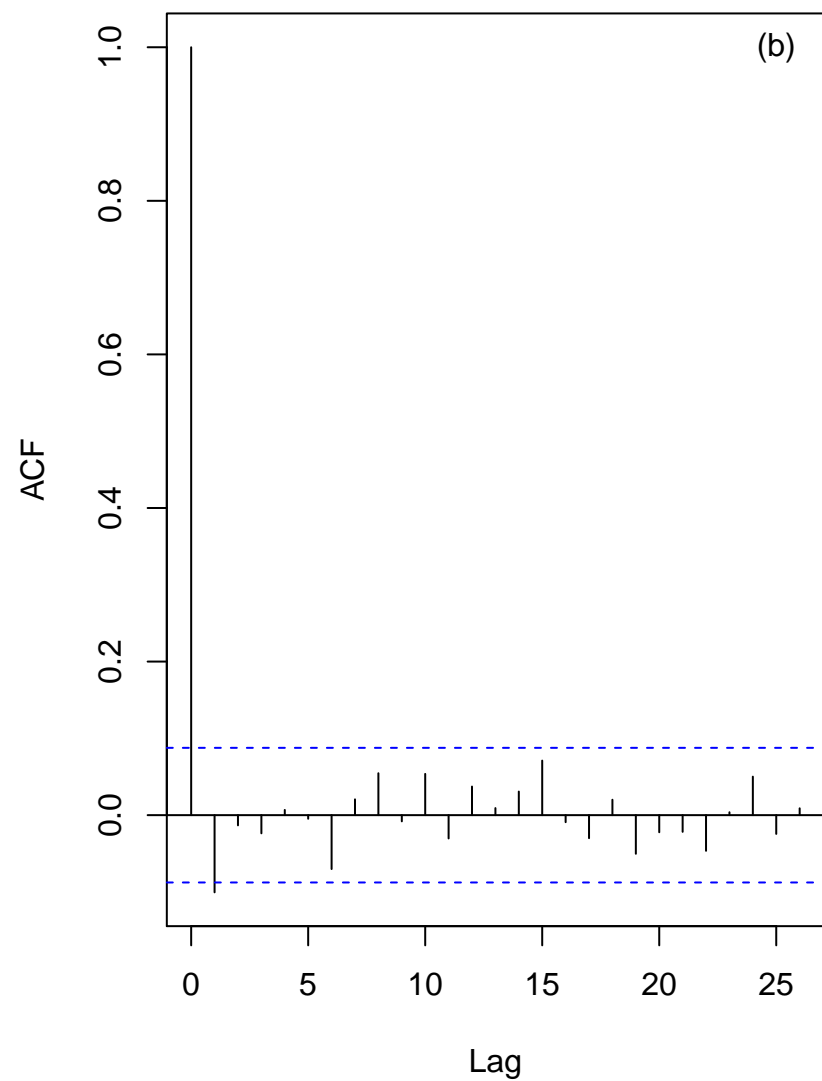

Supplement: S1 File — (ZIP) [file pone.0240046.s001.zip › DGQR estimation/residual.pdf]
